# Cerebral intraparenchymal extramedullary hematopoiesis in polycythemia vera

**DOI:** 10.1007/s13760-019-01196-2

**Published:** 2019-08-01

**Authors:** Jasmina Boban, Peter Kalhs, Majda M. Thurnher

**Affiliations:** 1 Department for Biomedical Imaging and Image-guided Therapy, University Hospital Vienna, Medical University of Vienna, Waehringer Guertel 18-20, 1090 Vienna, Austria; 2 Department for Internal Medicine, University Hospital Vienna, Medical University of Vienna, Waehringer Guertel 18-20, 1090 Vienna, Austria; 3 Present Address: Faculty of Medicine, University of Novi Sad, Hajduk Veljkova 3, Novi Sad, 21000 Serbia

Polycythemia vera (PV) is the most common myeloproliferative neoplasm, characterized by erythrocytosis, leukocytosis, and thrombocytosis and often associated with splenomegaly [1]. Neurological complications of PV are rare, but can include ischemic strokes, transient ischemic attacks (TIAs), and atypical TIAs [2].

Extramedullary hematopoiesis (EMH) is a compensatory physiological response of the organism to the failure of hematopoiesis at the bone marrow, which occurs commonly in patients with chronic hemolytic anemia or diseases with ineffective erythropoiesis [3]. Usually, extramedullary production of blood precursor cells occurs in the spleen, liver, or lymph nodes. However, cases with EMH in the adrenals, thymus, pleura, skin, gastrointestinal tract, and paranasal sinuses have been described [4]. Intracranial EMH is extremely rare, and only cases involving the dura mater have been described [3]. To the best of our knowledge, no cases of intracranial intraparenchymal EMH have been described.

A 48-year-old man with known polycythemia vera for 6 years presented with acute tinnitus. Six months before this, he was diagnosed with disease progression due to splenomegaly, anemia, leukocytosis (100,000 cells/μl,), and thrombocytopenia.

Magnetic resonance (MR) imaging of the brain was performed on a 1.5 T scanner (Philips Gyroscan Intera, NE), consisting of axial fluid attenuation inversion recovery (FLAIR), sagittal T1-weighted (T1WI), coronal T2-weighted (T2WI) MR images, diffusion-weighted imaging (DWI), and a post-contrast T1WI study in two planes. In the right temporal lobe, there was a T1WI isointense (Fig. 1b) and T2WI hypointense (Fig. 1c), intraparenchymal 2-cm-lesion, surrounded by a rim of T2W high-intensity perilesional edema. The lesion was adjacent to the right temporal horn. On DWI, the lesion showed a low signal. On post-contrast T1W images (Fig. 1d), there was a patchy, dot-like enhancement. There were no other brain lesions.

**Fig. 1:**
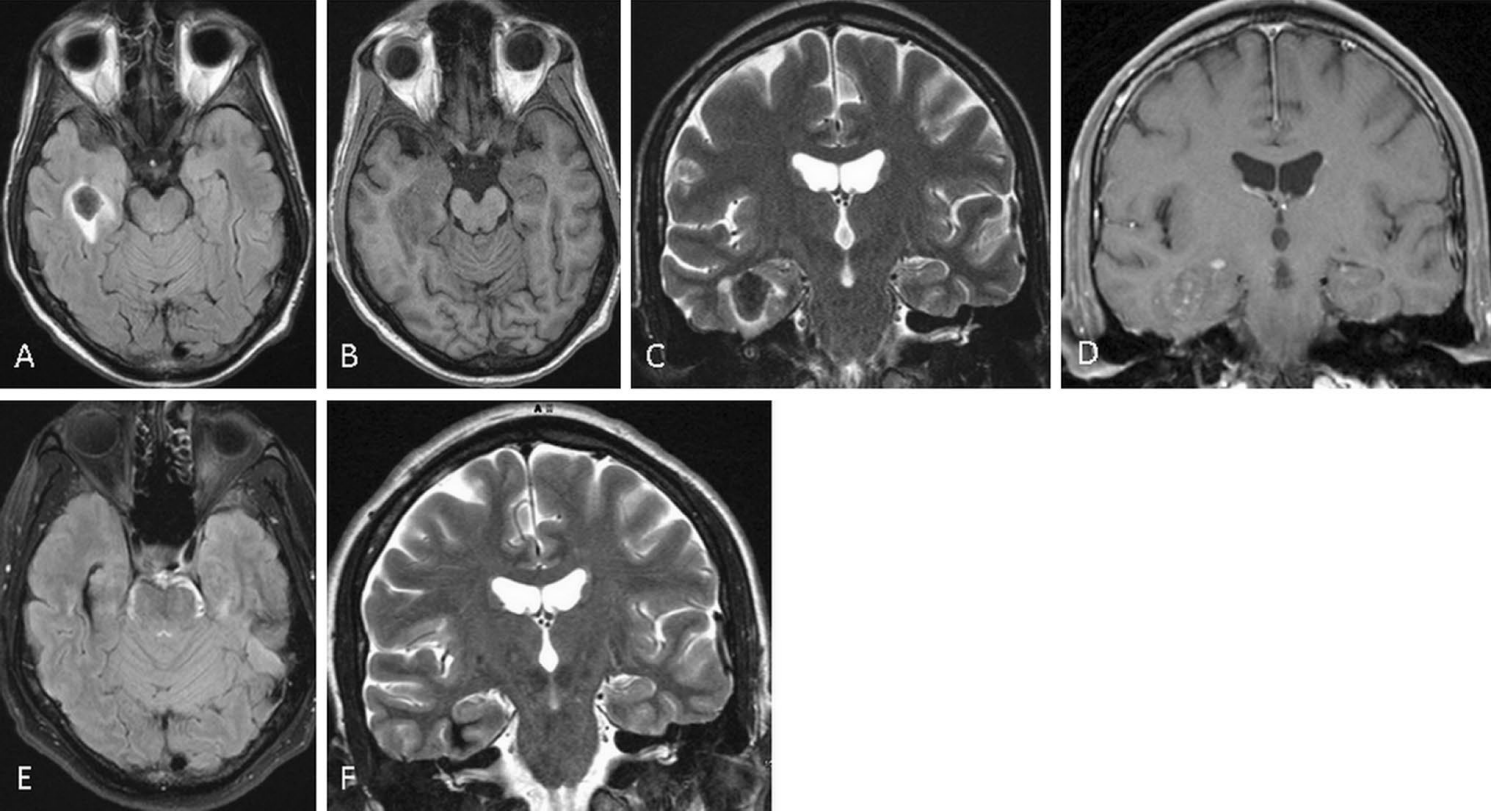
On the initial MR exam, there was a 2-cm FLAIR hypointense (**a**), T1WI isointense (**b**), T2 hypointense (**c**) lesion in the right temporal lobe adjacent to the temporal horn, with perilesional edema and patchy contrast enhancement (**d**). At the 6-month follow-up MR scan, there was only a small scar with hemosiderin deposits (**e**, **f**)

Elective splenectomy was performed 2 days later, and histology revealed extramedullary hematopoiesis. On a follow-up brain MR scan, 6 weeks after the initial exam, the lesion showed a significant reduction in size, with central T1W- and T2W hyperintensity and a dark peripheral rim. No edema and no contrast enhancement were detected. Six weeks later, on the last follow-up MR scan, there was only a non-enhancing linear scar with hemosiderin deposits (Fig. 1e, f). The patient’s neurological symptoms resolved completely. Three months after splenectomy, the patient underwent the first allogenic stem cell transplantation.

We present the first unique case of intracranial intraparenchymal EMH in a patient with polycythemia vera. PV is a myeloproliferative disorder, presenting with neurological complications mostly related to hypercoagulability and elevated hematocrit, such as ischemic strokes, TIAs, and atypical TIAs [2]. EMH is a compensatory, ectopic production of blood precursor cells in tissues other than bone marrow. Common sites of EMH are the liver, spleen, and lymph nodes. To date, cases of intracranial EMH have been described only in the form of extra-axial, epidural, or subdural, mass-like lesions [3]. The majority of the published cases were asymptomatic, presenting with symptoms only in cases of elevated intracranial pressure due to a tumor-like mass [5].

Imaging features of the lesions in our case were highly suggestive of extramedullary hematopoiesis; T1W isointensity and T2W hypointensity of the lesion were consistent with a high-cellularity lesion. Elevated diffusion excluded thromboembolic etiology. On SWI, the lesion showed susceptibility artifacts, suggesting the presence of blood products. Finally, there was a pattern of nonspecific, patchy enhancement, thus excluding lymphoma or sarcoid granuloma. The lesion showed reduction in size after treatment, resulting in a scar with a small hemosiderin deposition. Signs of radiological regression followed the clinical improvement of the patient.

One limitation of the study is the lack of histological proof of the diagnosis. However, imaging features of the lesion and the almost complete resolution of the lesion after splenectomy, in our opinion, exclude all potential differential diagnostic options.

The tinnitus in our patient can be explained by the extramedullary hematopoietic lesion in the temporal lobe that caused a disturbance in the network of the anterior cingulate cortex, the anterior insula, and the amygdala [6]. To conclude, although extremely rare, one should be aware of the possibility of intracranial intraparenchymal EMH in patients with myeloproliferative disorders, who present with neurological symptoms.

## References

[1] Spivak JL (2018). Polycythemia vera. Curr Treat Options Oncol.

[2] Michiels JJ, Berneman Z, Schroyens W (2006). Platelet-mediated erythromelalgic, cerebral, ocular and coronary microvascular ischemic and thrombotic manifestations in patients with essential thrombocythemia and polycythemia vera: a distinct aspirin-responsive and coumadin-resistant arterial thrombophilia. Platelets.

[3] Merchant R, Choudhari AJ, Verma M, Patkar DP, Doctor P (2018). Intracranial hematopoiesis in beta thalassemia: a case series. Ind J Pediatr.

[4] Ginzel AW, Kransdorf MJ, Peterson JJ, Garner HW, Murphey MD (2012). Mass-like extramedullary hematopoiesis: imaging features. Skelet Radiol.

[5] Eskazan AE, Ar MC, Baslar Z (2012). Intracranial extramedullary hematopoiesis in patients with thalassemia: a case report and review of the literature. Transfusion.

[6] Langguth B, Kreuzer PM, Kleinjung T, De Ridder D (2013). Tinnitus: causes and clinical management. Lancet Neurol.

